# A global microRNA screen identifies regulators of the ErbB receptor signaling network

**DOI:** 10.1186/s12964-015-0084-z

**Published:** 2015-01-29

**Authors:** Annabell Bischoff, Michaela Bayerlová, Michaela Strotbek, Simone Schmid, Tim Beissbarth, Monilola A Olayioye

**Affiliations:** Institute of Cell Biology and Immunology, University of Stuttgart, Allmandring 31, 70569 Stuttgart, Germany; Department of Medical Statistics, University Medical Center Göttingen, 37099 Göttingen, Germany

**Keywords:** MicroRNA/miRNA, ErbB3/HER3, Heregulin, PI3K-Akt pathway, Breast cancer

## Abstract

**Background:**

The growth factor heregulin (HRG) potently stimulates epithelial cell survival and proliferation through the binding of its cognate receptor ErbB3 (also known as HER3). ErbB3-dependent signal transmission relies on the dimerization partner ErbB2, a receptor tyrosine kinase that is frequently overexpressed and/or amplified in breast cancer cells. Substantial evidence suggests that deregulated ErbB3 expression also contributes to the transformed phenotype of breast cancer cells.

**Results:**

By genome-wide screening, we identify 43 microRNAs (miRNAs) that specifically impact HRG-induced activation of the PI3K-Akt pathway. Bioinformatic analysis combined with experimental validation reveals a highly connected molecular miRNA-gene interaction network particularly for the negative screen hits. For selected miRNAs, namely miR-149, miR-148b, miR-326, and miR-520a-3p, we demonstrate the simultaneous downregulation of the ErbB3 receptor and multiple downstream signaling molecules, explaining their efficient dampening of HRG responses and ascribing to these miRNAs potential context-dependent tumor suppressive functions.

**Conclusions:**

Given the contribution of HRG signaling and the PI3K-Akt pathway in particular to tumorigenesis, this study not only provides mechanistic insight into the function of miRNAs but also has implications for future clinical applications.

**Electronic supplementary material:**

The online version of this article (doi:10.1186/s12964-015-0084-z) contains supplementary material, which is available to authorized users.

## Background

ErbB receptors play a well-established role in various types of cancer, including those of the breast. The ErbB receptors comprise four members: epidermal growth factor receptor EGFR/ErbB1, ErbB2/HER2, ErbB3/HER3 and ErbB4/HER4. In breast cancer, ErbB2/HER2 is found to be amplified and/or overexpressed in up to 30% of patients, correlating with poor prognosis [1,2]. Although ErbB2/HER2 function can successfully be blocked with antibodies such as Herceptin/Trastuzumab, a subset of tumors is found to be non-responsive or tumor cells acquire resistance during therapy. In this context, ErbB3/HER3 has attracted growing attention because upregulation of signaling through this receptor plays an important role in the resistance to targeted therapies. This can be explained by the very efficient coupling of ErbB3 to the phosphatidylinositol 3-kinase (PI3K)/Akt survival pathway [1,2].

Signaling pathways activated by ErbB receptors include the mitogen activated protein kinase (MAPK) and PI3K pathways, which stimulate cellular responses such as survival, proliferation, and migration. Ligand binding to ErbB receptors induces the formation of homo- and heterodimers and activation of the intrinsic kinase domain, resulting in the phosphorylation of a set of tyrosine residues within their cytoplasmic tails. These phosphorylated residues serve as docking sites for Src homology 2 and phosphotyrosine-binding domain containing proteins, the recruitment of which leads to the activation of specific intracellular signaling pathways [3,4]. The pathways activated are dependent upon the individual ErbB receptors due to their ability to bind distinct effector proteins. Heregulin (HRG) specifically binds to ErbB3 and ErbB4, inducing heterodimerization mainly with ErbB2. Although ErbB2 has no direct ligand, it readily dimerizes with the other ErbB receptors due to its constitutively active conformation, as revealed by structural analyses [5]. ErbB3 is unique in that it has an impaired kinase domain lacking catalytic function, but in a heterodimer with a signaling competent ErbB family member, ErbB3 is transphosphorylated, thus serving as a signaling platform. The presence of six binding sites for p85, the regulatory subunit of PI3K, within the ErbB3 cytoplasmic tail explains the strong activation of this pathway by ErbB3-containing heterodimeric ErbB receptor complexes [6]. The production of PIP3 by PI3K recruits Akt to the plasma membrane via its pleckstrin homology domain, where Akt is then activated by phosphorylation on threonine 308 by phosphoinositide-dependent kinase 1 and on serine 473 by mTOR complex 2 or DNA-PK, the latter phosphorylation known to be required for full kinase activity [7].

microRNAs (miRNAs) are 17-nt to 24-nt long non-coding RNAs that regulate gene expression in metazoans [8]. miRNAs act by partial complementary binding to their target mRNAs usually within the 3′-untranslated region (3′UTR), resulting in translational repression and/or mRNA degradation [9]. Microarray and proteomic experiments have demonstrated the impact of a single miRNA on fine-tuning expression of a hundred of targets [10]. Fundamental biological cellular processes such as development, proliferation, tumorigenesis and apoptosis are now known to be regulated by miRNAs. Furthermore, miRNAs are implicated in various diseases including the development of cancer, making them attractive targets for therapeutic intervention or as diagnostic markers [11,12]. In recent years, miRNA profiling studies undertaken in different tumor types have identified sets of miRNAs that have altered expression in tumor versus normal tissue. While certain upregulated miRNAs, so-called oncoMirs, such as miR-155 and the miR17-92 cluster have been characterized to possess transforming potential, there are also examples of miRNAs with inherent tumor suppressive activity, such as the let-7 family, that are downregulated in cancer cells [11,12].

miRNAs that impact ErbB signaling are of special interest due to the major contribution of this pathway to cancer progression. miR-7 was one of first miRNAs identified to simultaneously target EGFR and downstream signaling molecules including Raf1 [13,14]. Furthermore, miR-125a and miR-125b* were shown to co-target ErbB2 and ErbB3, which was accompanied by the suppression of MAPK and PI3K signaling pathways [15]. miR-199a, on the other hand, contributes to enhanced HRG-induced ErbB2-ErbB3 signaling. This miRNA exerts its oncogenic effect by reducing the protein level of Necl-2, which is involved in receptor dephosphorylation via PTPN13 [16,17]. Moreover, upregulation of miR-21, which targets the tumor suppressor and lipid phosphatase PTEN and therefore promotes sustained ErbB receptor signaling, was detected in cells with acquired Herceptin resistance [18]. These examples of miRNA-mediated regulation of the ErbB signaling network at different levels illustrate the importance of miRNA regulation and emphasizes their potential for prognostic and therapeutic purposes.

Here, we performed a genome-wide miRNA screen in the breast cancer cell line MCF7 based on Akt phosphorylation as a read-out to investigate the extent by which miRNAs modulate the ErbB receptor signaling pathway. Our data analysis identifies 43 miRNAs that specifically regulate HRG-induced Akt activation, either positively or negatively, and reveals the complexity of coordinated miRNA-target interactions within the ErbB signaling pathway. We further validate four miRNAs with potential tumor suppressive function as novel regulators of ErbB3 transcript and protein levels, the expression of which is shown to block HRG-dependent proliferation. Our findings thus give mechanistic insight into miRNA function and have implications for future clinical applications.

## Results

### Screen establishment: analysis of heregulin-induced signaling by miR-149

**Figure 1:**
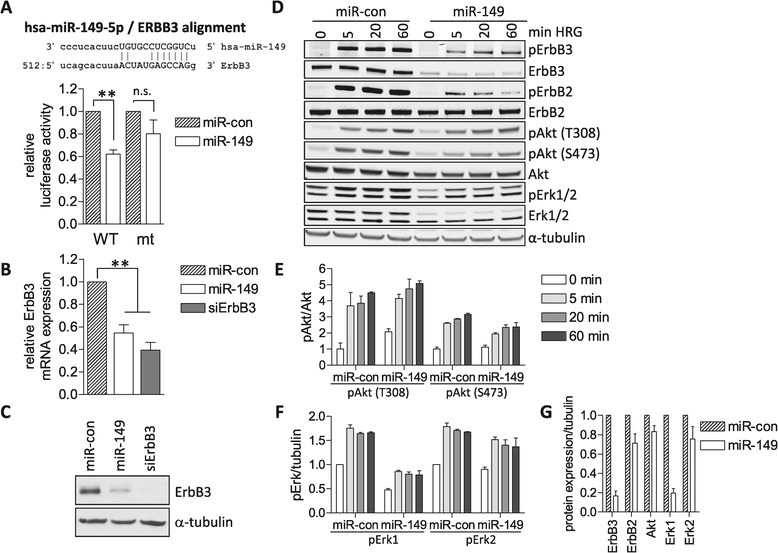
**miR-149 suppresses heregulin signaling. (A)** miR-149 recognition site in the ErbB3 3′UTR (upper panel). HEK293T cells were co-transfected with miR-con or miR-149 along with a luciferase reporter containing the wild-type (WT) or mutated ErbB3-UTR lacking the miR-149 seed region (mt; Δ527-533). The next day, cells were lysed and luciferase activity measured and normalized to the activity of the co-expressed Renilla reporter (lower panel). Data correspond to the mean ± SEM of four independent experiments performed with triplicate samples. Data were analyzed by one way Anova followed by Tukey’s multiple comparison test (**p < 0.01). **(B)** MCF7 cells were transfected with an ErbB3-specific siRNA pool (siErbB3), miR-con or miR-149, respectively. Three days post transfection, RNA was extracted and ErbB3 levels were determined by qRT-PCR. Values were normalized to GAPDH. Data are shown as the mean ± SEM of three independent experiments and analyzed by one-way Anova followed by Tukey’s multiple comparison test (**p < 0.01). **(C, D)** MCF7 cells were transfected with miRNAs and siRNAs as indicated and analyzed three days later. **(C)** Cells were lysed and ErbB3 expression analyzed by immunoblotting. Tubulin was detected as a loading control. **(D)** Cells were left unstimulated (0 min) or stimulated with 10 ng/ml HRG for the indicated times prior to lysis. Cell lysates were analyzed by immunoblotting using the indicated antibodies **(E-G)** Western Blot signals from two independent experiments were quantified by ImageJ. pAkt signals were normalized to total Akt (E), whereas phosphorylated Erk signals were normalized to tubulin because total Erk levels were strongly affected by miRNA expression (see G; signals at 0 min HRG). The unstimulated control was set to 1. The mean intensities ± SEM are shown.

To screen for miRNA regulators of the ErbB receptor pathway we chose MCF7 cells, a well characterized luminal breast cancer cell line that expresses all four ErbB receptors and is responsive to HRG. To establish the screen and define the magnitude of miRNA-mediated effects, we first sought a control miRNA that directly targets ErbB receptors. Because miR-125a, previously reported to co-target ErbB2 and ErbB3 [15], did not affect expression of these receptors in MCF7 cells (data not shown), we validated miR-149 as a potential miRNA control. In a previous study, this tumor-suppressive miRNA was predicted to affect the ErbB pathway based on KEGG pathway analysis [19]. Using the miRanda prediction algorithm (http://www.microrna.org) we identified a conserved binding site for hsa-miR-149-5p (miR-149) within the 3′-UTR of the ErbB3 sequence (Figure 1A). Using a vector containing the 3′UTR of ErbB3 cloned downstream of the luciferase cDNA [15], we confirmed that miR-149 directly targets ErbB3. Coexpression of a miR-149 mimic reduced luciferase activity in cell lysates compared to the activity measured in lysates from cells co-expressing the control miRNA (Figure 1A). Deletion of the seven nucleotides complementary to the miR-149 seed region from the potential recognition motif in the ErbB3 3′UTR partially restored luciferase activity, indicating that miR-149 blocks luciferase expression by directly binding the ErbB3 3′UTR (Figure 1A). Transient transfection of MCF7 cells with miR-149 followed by qRT-PCR analysis and immunoblotting revealed potent suppression of ErbB3 transcript and protein levels, respectively, compared with those in miRNA-control transfected cells (Figure 1B,C). As a positive control, an ErbB3-specific siRNA pool was used, which efficiently silenced ErbB3 expression (Figure 1B,C).

Having established ErbB3 as a target of miR-149, we next investigated the impact of miR-149 on HRG-induced phosphorylation kinetics by immunoblotting of the receptors and the downstream kinases Erk1/2 and Akt as readouts for PI3K and MAPK pathways, respectively. In agreement with the data shown in Figure 1C, miR-149 expression decreased ErbB3 protein levels, thereby impairing HRG-induced phosphorylation and activation of ErbB3 itself and its dimerization partner ErbB2 (Figure 1D). This potent suppression of ErbB2/3 phosphorylation was accompanied by reduced Erk1/2 phosphorylation and modestly reduced Akt(S473) phosphorylation (Figure 1D-F). In the case of Akt(T308) phosphorylation, the levels in HRG-stimulated control and miR-149 expressing cells were similar, however, in the latter cells the fold induction was reduced due to increased basal Akt(T308) phosphorylation (Figure 1D,E), possibly resulting from the compensatory activation of feedback mechanisms. Apart from its effect on ErbB3, miR-149 expression also reduced Erk1 protein levels, whereas only subtle changes in Erk2, Akt and ErbB2 were observed (Figure 1D,G). Because miRNAs often co-regulate several targets within a specific signaling pathway, it is possible that miR-149 also regulates Erk1 post-transcriptionally; alternatively, miR-149 may affect Erk1 expression indirectly.

**Figure 2:**
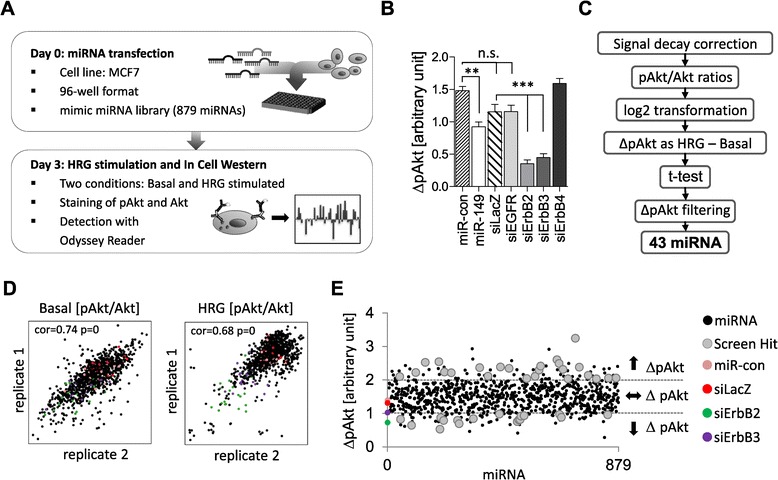
**Genome-wide miRNA screen. (A)** Workflow of the screening procedure. MCF7 cells were transiently transfected in a 96-well format with a human mimic miRNA library comprising 879 human miRNAs. Three days post transfection, cells were either left untreated (basal) or stimulated with heregulin (HRG) for one hour followed by the detection of total Akt and phosphorylated Akt(pT308) levels by In-Cell Western. **(B)** MCF7 cells were transfected with the indicated miRNAs and siRNAs and subjected to In-Cell Western as described in **(A)**. Akt activation after HRG stimulation (ΔpAkt) was calculated as described in the Methods section. One representative experiment performed with triplicate samples is shown. The data was analyzed by one way Anova followed by Tukey’s multiple comparison test (**p < 0.01, ***p < 0.001). **(C)** Workflow of the bioinformatic analysis of the screen data. For each replicate, the ratio of pAkt/Akt signal intensity and the difference between untreated and stimulated samples were calculated. miRNAs that significantly (*p* < 0.05) altered Akt activity and had values of ΔpAkt < 1 or ΔpAkt > 2 for both replicates were considered as screen hits. **(D)** Correlation of the ratios of phosphorylated Akt and total Akt signal intensities (pAkt/Akt) for the basal (left) and HRG-stimulated (right) condition. **(E)** Screen data visualization: Akt activation upon HRG stimulation (ΔpAkt) for each miRNA is plotted. The mean of the two replicates for each miRNA is presented in ascending order.

For screening purposes, we transferred the analysis of HRG signaling to a 96-well format using an In-Cell Western protocol (Figure 2A). This method is based on the direct antibody staining of cells, and when combined with IRDye-labeled secondary antibodies and the Odyssey scanning system, it yields quantitative data and enables the simultaneous detection of two signals in a single well. Testing of the different phosphospecific antibodies used in Figure 1D by In-Cell Western revealed that the pAkt(T308)- and pErk-specific antibodies gave rise to specific and sustained signals upon HRG stimulation with kinetics corresponding to those of the Western blot (Additional file 1: Figure S1), whereas the pAkt(S473)-specific antibody did not yield a specific signal under these conditions (data not shown). Considering the greater dynamic range of the pAkt(T308) signal upon stimulation of cells with different HRG concentrations (Additional file 1: Figure S1), we selected the pAkt(T308) antibody, an intermediate HRG concentration (10 ng/ml) and stimulation of cells for 1 hour for our high-throughput screen. Next, MCF7 cells were transfected with a control miRNA, miR-149, a control siRNA (siLacZ) and siRNAs against all members of the ErbB family, stimulated with HRG followed by co-staining with pAkt and Akt antibodies, respectively. In parallel, unstimulated cells were stained to determine basal Akt activity. pAkt/Akt ratios were determined for each sample and the basal values were then subtracted from the HRG-stimulated ones, yielding ΔpAkt (see Methods for details). Compared with the controls, ErbB2 and ErbB3 knockdowns almost completely abolished HRG-induced Akt activation, whereas ErbB1 and ErbB4 had minimal effects (Figure 2B), confirming that ErbB2/3 is the relevant signal heterodimer in this setting. Compared to the miRNA control, ectopic expression of miR-149 reduced ΔpAkt by ~40% (Figure 2B), in accordance with the reduced fold induction observed by Western blot (Figure 1E), demonstrating that miRNA-mediated modulation of ErbB receptor signaling can be quantified using this method.

### Genome-wide miRNA screening for regulators of HRG-induced Akt activation

In-Cell Western screening of a miRNA mimic library comprising 879 miRNAs (Additional file 2: Table S1) was performed as detailed in Figure 2A. The screen was repeated to obtain a second independent data set for both the basal and HRG-stimulated condition. Screen data were then normalized by determining pAkt/Akt ratios for all replicate samples (see Additional file 2: Table S2 for raw data). For quality control, the replicates were correlated yielding an average Pearson’s coefficient of 0.74 (basal) and 0.68 (HRG), respectively (Figure 2D). HRG-induced Akt activation was determined and plotted for all miRNAs (ΔpAkt; Figure 2C). The majority of miRNAs had no effect on ΔpAkt values and clustered in the range of the negative controls miR-con and siLacZ (Figure 2E; Additional file 1: Figure S2). By contrast, ΔpAkt values for the positive controls miR-149, siErbB2 and siErbB3 were ≤1 (Figure 2E, Additional file 1: Figure S2) and therefore used to define a cut-off: Only those miRNAs were considered as screen hits for which ΔpAkt changes were significant and either below 1 or greater than 2 (Figure 2C,E). 43 miRNAs met these criteria, 19 of which significantly reduced (ΔpAkt < 1) and 24 of which significantly increased HRG-induced Akt activation (ΔpAkt > 2).

**Figure 3:**
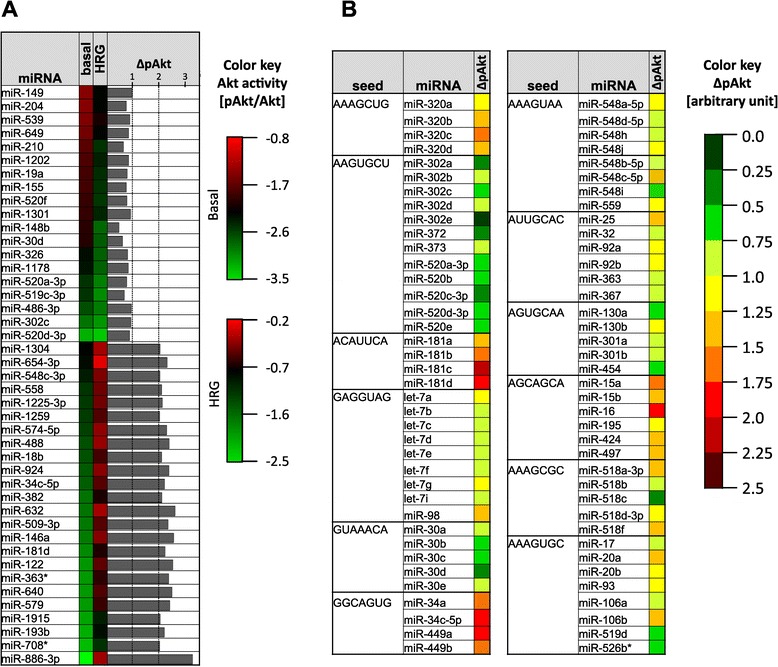
**Analysis of the screen data. (A)** Screen hits depicted in list format. Mean values of the pAkt/Akt ratios for the basal and HRG-stimulated condition are shown, with green indicating a reduction and red an increase compared to the mean of miR-con (black). ΔpAkt values (mean of the two replicates) for each miRNA screen hit is shown as a bar chart. **(B)** miRNAs were clustered according to their seed region and for each miRNA ΔpAkt is visualized as a color-coded value. All seed families with more than 3 members are listed. Values correspond to the mean of the two replicates.

In Figure 3, the ΔpAkt values as well as the basal and HRG-stimulated pAkt/Akt ratios are depicted for the miRNA screen hits. Red coloring indicates an increase, green coloring a decrease of the pAkt/Akt level compared to that in miR-con expressing cells. This reveals that those miRNAs that enhance HRG-stimulated pAkt/Akt levels generally suppress basal pAkt/Akt levels. This is most pronounced for miR-886-3p, for which the greatest difference in HRG-induced Akt activation is observed (ΔpAkt = 3.25). In the case of miR-1304 and miR-654-3p, basal pAkt/Akt levels were unaffected, thus, the increase in ΔpAkt was specifically due to enhanced Akt activity upon HRG stimulation. For those miRNAs that negatively affected ΔpAkt, there was no uniform trend regarding basal pAkt/Akt levels, as some miRNAs such as miR-204 enhanced, whereas others, e.g. miR-520a-3p, reduced basal pAkt/Akt levels. Regardless of the effect on basal Akt activity, all miRNA hits in this category attenuated Akt activation in response to HRG. This is best reflected by miR-148b, for which the lowest ΔpAkt value was obtained, indicating almost complete suppression of HRG-induced Akt activation. Furthermore, a general reduction of pAkt/Akt levels for the basal and HRG-stimulated situation is seen for miR-520a-3p, miR-519c-3p, miR-485-3p, miR-302c and miR-520d-3p. Notably, three of these miRNAs possess the same seed region, which is critical for target recognition. The similar ΔpAkt values obtained for the different seed family members underscores the reliability of the screen and suggests that the effects of these miRNAs are mediated by common targets (Figure 3B).

### Construction of an ErbB/Akt signaling network with integrated miRNA-target information

For network construction we used data from the Reactome database [20] and identified 24 pathways connected to ErbB/Akt signaling. These pathways were parsed into R using r-package rBiopaxParser [21] as directed graphs. Next, 24 graphs were merged into a single signaling network, comprising 312 nodes representing genes, and 3582 edges representing activation or inhibition effects. Because some of the genes are components of protein complexes, to simplify the network, we merged these gene-nodes into protein-complex-nodes. This was done for PIK3 (PIK3CA, PIK3R1), mTORC1 (AKT1S1, MLST8, MTOR) and mTORC2 (RICTOR, MLST8, MTOR), finally yielding 309 nodes and 3542 edges. To acquire miRNA-target information for the 43 screen hit miRNAs, we searched 3 databases with computationally predicted miRNA targets: MicroCosm Targets release v5 [22], miRDB v4.0 [23] and microRNA.org August 2010 release [24]. For 3 miRNAs (miR-1304, miR-1259, miR-1915) no targets were found in these databases. The remaining 40 miRNAs were divided into two groups: a first group with negative effects on ΔpAkt (19 miRNAs), and a second group with positive effects on ΔpAkt (21 miRNAs). We pooled miRNA – target information from all three databases and identified 298 target genes in the ErbB/Akt signaling network (see Additional file 2: Table S3). Next, we separately connected both groups of miRNAs with the network by 959 and 750 miRNA – target gene edges for the first and second miRNA group, respectively. This resulted in the construction of two miRNA-ErbB/Akt signaling networks. To visualize these networks, the target genes in each network were ranked by the number of targeting miRNAs and the top target genes were then selected. For the network with miRNAs that negatively affected ΔpAkt, a sub-network comprising the top 14 genes targeted by 9 or more miRNAs was extracted with corresponding miRNAs. In the case of the network with positively acting miRNAs, a sub-network of top 17 genes, targeted by 6 or more miRNAs, was extracted with corresponding miRNAs (Additional file 1: Figures S4 and S5). In agreement with the opposing effects on ΔpAkt, the target genes identified for negatively and positively acting mRNAs differed, showing little overlap.

**Figure 4:**
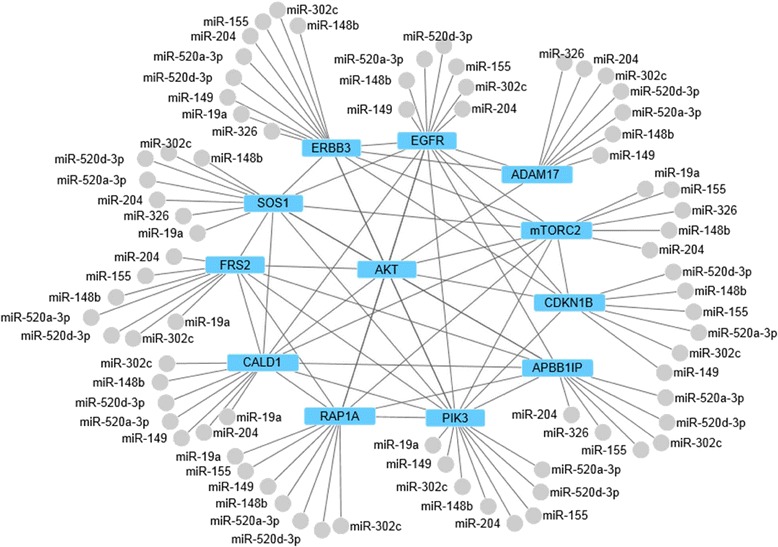
**miRNA-protein interaction network for negative regulators of HRG signaling.** The 14 most frequently targeted genes plus Akt are shown. The edges are based on signaling data of Reactome and protein-protein interactions of STRING databases. Only those miRNAs that co-target ErbB3 are depicted.

Next, we focused our attention on those miRNAs that reduced Akt activation because their effect can be explained by the direct targeting of key players within the ErbB-Akt pathway. Interestingly, ErbB3 and PIK3 were among the most frequently predicted target genes. To investigate in more detail the potential co-regulation of these genes, we created a miRNA-protein interaction graph that integrated additional information on protein-protein interactions from the STRING database [25] and comprised only directly connected proteins and miRNAs predicted to target ErbB3, namely miR-520a-3p, miR-520d-3p, miR-302c, miR-19a, miR-148b, miR-204, miR-155, miR-149, miR-326 (Figure 4 and Additional file 1: Figure S3). Intriguingly, all nine miRNAs, of which only 520a-3p, 520d-3p and 302c were members of the same seed family, had largely overlapping target spectra. Apart from ErbB3 and PIK3, genes such as RAP1A, a member of the Ras GTPase family, mTOR complex 2, the Ras GDP exchange factor SOS1 and the metalloproteinase Adam17/Tace were predicted as targets. This indicates that this subset of miRNAs negatively regulates HRG-induced Akt activation by targeting the PI3K pathway at multiple levels, thereby multiplying the effects caused by the post-transcriptional suppression of single pathway components.

### miR-148b, miR-149, miR-326 and miR-520a reduce ErbB3 expression and dampen HRG-induced MAPK and PI3K signaling

**Figure 5:**
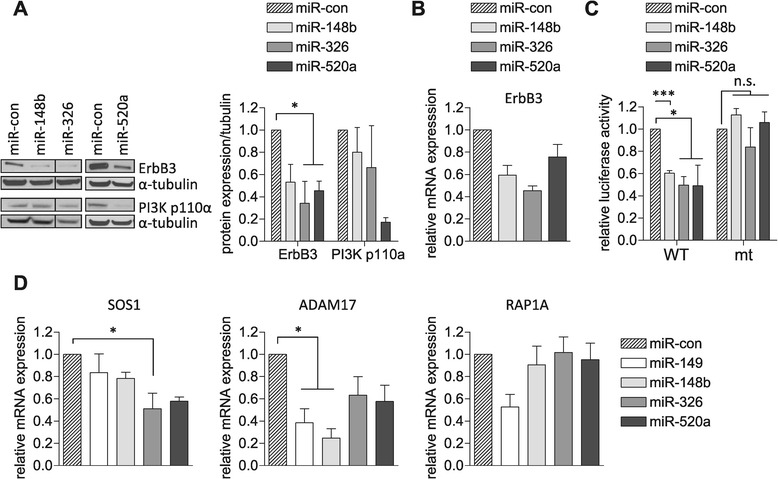
**miR-148b, miR-326 and miR-520a affect the ErbB signaling pathway at multiple levels. (A)** MCF7 cells were transfected with miR-con, miR-148b, miR-326 and miR-520a (miR-520a-3p), respectively. Three days after transfection, cells were lysed and lysates analyzed by immunoblotting using ErbB3 and PI3K p110α specific antibodies. Tubulin was detected to confirm equal loading. A representative Western blot is shown; the left panels are from the same blot. Signals were quantified by ImageJ and normalized to those of tubulin. Data are shown as the mean ± SEM of three to five independent experiments and analyzed by one way Anova followed by Tukey’s multiple comparison test (* *p* < 0.05). **(B)** MCF7 cells were transfected with the indicated miRNAs. Two days post transfection, RNA was extracted and ErbB3 levels were determined by qRT-PCR. Values were normalized to GAPDH. Data are shown as the mean ± SEM of two independent experiments. **(C)** HEK293T cells were co-transfected with the indicated miRNAs and the different luciferase reporter constructs containing wild-type (WT) ErbB3 mRNA sequences or those lacking the respective miRNA seed regions (mt) (see Methods and Additional file 1: Figure S7). The next day, cells were lysed and luciferase activity measured and normalized to the activity of the co-expressed Renilla reporter. Data correspond to the mean ± SEM of three independent experiments performed with at least triplicate samples. Data analysis: one way Anova followed by Tukey’s multiple comparison test (*p < 0.05, ***p < 0.001). **(D)** MCF7 cells were transfected with the indicated miRNAs. Three days post transfection, RNA was extracted and SOS1, ADAM17 and RAP1A levels were determined by qRT-PCR. Values were normalized to GAPDH. Data are shown as the mean ± SEM of three independent experiments and analyzed by one way Anova followed by Tukey’s multiple comparison test (*p < 0.05).

We next sought to validate ErbB3 as a target for select miRNA screen hits: miR-148b gave rise to the strongest reduction of ΔpAkt; miR-520a-3p was chosen as a representative of the 520/302 seed family; miR-326 was the only miRNA for which no co-regulation of ErbB3 and PIK3 was predicted. To this end, MCF7 cells were transiently transfected with the non-targeting control miRNA (miR-con), miR-148b, miR-326 and miR-520a-3p, followed by Western blot analyses of total cell lysates. In line with the target prediction, reduced ErbB3 expression was confirmed for miR-148b, miR-326, and miR-520a-3p (Figure 5A). By contrast, downregulation of the catalytic subunit of PIK3 (PI3K p110α) was only evident in cells overexpressing miR-520a-3p (Figure 5A). qRT-PCR analyses further confirmed reduced ErbB3 transcript levels (Figure 5B), suggesting that the ErbB3 mRNA is targeted by miR-148b, miR-326, and miR-520a-3p. To test this directly, luciferase expression vectors with mRNA regions of ErbB3 containing the predicted binding sites of these miRNAs were generated as detailed in the Methods section. Co-transfection of miR-148b, miR-326 or miR-520a-3p with the different luciferase expression vectors suppressed luciferase activities, which was fully restored by deletion of the respective seed regions from the predicted binding sites (Figure 5C, Additional file 1: Figure S7), providing evidence for the direct targeting of the ErbB3 mRNA by miR-148b, miR-326 and miR-520a-3p.

To explore the extent by which these miRNAs triggered degradation of additional targets associated with the ErbB signaling network, we further assessed SOS1, Adam17, and Rap1a mRNA levels by qRT-PCR. These experiments revealed miRNA-induced downregulation of Adam17 and SOS1 transcripts in all cases (Figure 5D), in accordance with the target prediction. However, Rap1a transcript levels were only suppressed by miR-149 (Figure 5D). It is possible that miR-148b and miR-520a-3p regulate Rap1a expression by translational inhibition rather than degradation, however, it should also be noted that *in silico* prediction algorithms give rise to false positives and therefore any candidates must be experimentally validated as genuine targets. Nevertheless, our analysis confirms the simultaneous suppression of a number of ErbB pathway molecules by the miRNA screen hits at the transcript level.

**Figure 6:**
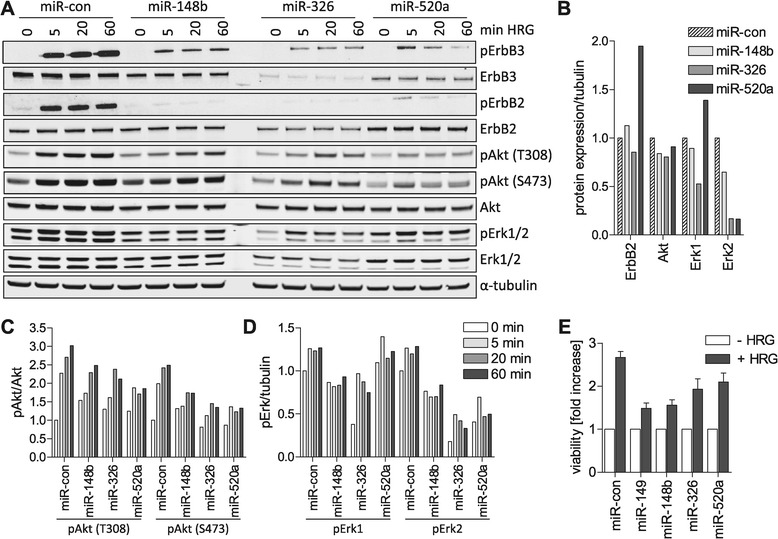
**miRNA inhibition of HRG-dependent signaling and cell viability.** MCF7 cells were transfected with the indicated miRNAs. **(A)** Three days after transfection, cells were either left untreated (0 min) or stimulated with 10 ng/ml HRG for the indicated times prior to lysis. Equal amounts of cell lysate were analyzed by immunoblotting using antibodies specific for ErbB3(pY1289), ErbB2(pY1221/1222), Akt(pT308), Akt(pS473), and Erk1/2(pT202/pY204). Membranes were further probed with antibodies that detect the total level of these proteins. Tubulin was detected to confirm equal loading. **(B-D)** Western Blot signals from **(A)** as a representative experiment were quantified by ImageJ. pAkt signals were normalized to total Akt **(C)**, whereas phosphorylated Erk signals were normalized to tubulin **(D)** because total Erk levels were strongly affected by miRNA expression (see B; signals at 0 min HRG). The unstimulated control was set to 1. **(E)** One day after transfection, cells were plated into 96-well plates and grown in medium containing 0.5% FCS (− HRG) and 0.5% FCS supplemented with 5 ng/ml HRG (+ HRG), respectively. Five days later, cells were fixed and stained with crystal violet and the absorbance at 550 nm was measured. Data were normalized to the basal growth in 0.5% FCS. The mean ± SEM of 2–4 independent experiments is shown.

*miR-148b, miR-149, miR-326 and miR-520a reduce HRG-induced signaling and viability.* We next investigated how the selected miRNAs affected ErbB2/3 receptor activation and downstream signaling by immunoblotting of cell lysates derived from HRG-stimulated cells. In agreement with Figure 5A, the expression of miR-148b, miR-326 and miR-520a-3p reduced ErbB3 protein and phosphorylation levels, which was accompanied by reduced ErbB2, Akt and Erk1/2 phosphorylation (Figure 6A, C-D). In addition, overexpression of miR-148b and miR-520a-3p reduced Erk2 protein levels, whereas miR-326 affected both, Erk1 and Erk2 (Figure 6A,B). Erk1/2 are not predicted targets for these miRNAs based on perfect base pairing of the seed region, however, increasing evidence suggests that nucleotides other than the seed region contribute to efficient miRNA targeting. Furthermore, ErbB2 and Erk1 levels were increased by miR-520a-3p expression, possibly resulting from compensatory mechanisms. Taken together, these experiments confirm that miR-148b, miR-326 and miR-520a-3p reduce ErbB3 expression and severely impact HRG-induced ErbB receptor downstream signaling. HRG signaling was also negatively affected by miR-149 and miR-520a-3p expression in SKBR3 cells, a breast cancer cell line with ErbB2 amplification. In both cases, miRNA expression suppressed ErbB3 expression and reduced HRG-induced Akt and Erk phosphorylation (Additional file 1: Figure S8A), however, in the case of miR-148b no suppression was observed (data not shown). Note that miR-149 expression appears to favor HRG-induced ErbB3 degradation in these cells, demonstrating that the precise signaling response differs in different cell lines. A recent study by the Sorger lab on growth factor signaling in different cell lines supports the view that growth factor responses across different breast cancer cell lines are diverse, even within the same subtype [26]. This diversity was found to arise from the variation in the abundance of the receptors themselves and in the abundance and activity of downstream signaling molecules.

HRG is known to support the viability of breast cancer cells. To assess the impact of the selected negative miRNA screen hits in a biological assay, we measured the viability of MCF7 cells ectopically expressing miR-148b, miR-149, miR-326 and miR-520a-3p in the presence of HRG. In control cells, viability was increased 2.8 fold in medium containing 0.5% FCS and HRG compared with medium supplemented with 0.5% FCS only (Figure 6E). In the presence of HRG, the viability of cells was reduced by all miRNAs (Figure 6E). Furthermore and consistent with the inhibition of HRG signaling, miR-149 and miR-520a-3p suppressed the migration of SKBR3 cells in Transwell assays containing HRG in the bottom chamber (Additional file 1: Figure S8B). These data provide support for the inhibition of HRG-induced biological responses by these miRNAs and their potential tumor suppressive function.

## Discussion

The tight control of HRG responses is of fundamental importance for proper cell function as, on the one hand, HRG-induced signaling is essential for the morphogenesis and differentiation of the mammary gland [27] and, on the other hand, up-regulation of HRG signaling is sufficient to drive malignant transformation and the development of tumors in the breast [28]. Here, we identified by genome-wide screening miRNAs that modulate HRG-induced Akt activation in MCF7 breast cancer cells. By setting a stringent cut-off requiring effects to be at least as potent as those of miR-149, we identified 43 miRNAs that specifically altered HRG-induced Akt activation, with 24 increasing and 19 decreasing Akt activation. The similar results obtained for individual miRNA seed family members underscore the importance of this region in target selection and prove the robustness of our screen. In addition to miR-149, nine miRNA screen hits that suppressed HRG-induced Akt activation were predicted to target the ErbB signaling network at the level of ErbB3 concomitant with key downstream effector proteins. Three miRNAs, namely miR-148b, miR-326 and miR-520a-3p as a representative of the miR-520 seed family, were validated to reduce both ErbB3 transcript and protein levels, indicating that these miRNAs, similarly to miR-149, target ErbB3 directly.

Despite its ligand-independent active conformation, ErbB2-induced transformation of breast cancer cells is dependent on ErbB3 [29,30]. Moreover, due to the potent activation of the PI3K survival pathway, ErbB3 plays an important role in the responsiveness of cancer cells to targeted therapies. Breast cancer cells were shown to upregulate ErbB3 via multiple mechanisms to escape pharmacological EGFR inhibition [31], and in lung cancer cells, autocrine activation of ErbB3 caused resistance to the EGFR-specific small molecule inhibitor Gefitinib [32]. miRNA alterations may thus modulate the efficacy of such treatments by impacting ErbB3 expression. In support of this hypothesis, drug-resistant MCF7 cells showed downregulation of miR-326 and ectopic re-expression sensitized cells to doxorobucin [33]. Additionally, in SKBR3 cells, miR-205, previously shown to target ErbB3, blocked Akt activity and increased the response to the pharmacological ErbB inhibitors Gefitinib and Lapatinib [34].

Because miRNAs tend to have moderate effects on the expression of a single gene, the simultaneous targeting of several genes contained within a functional signaling unit appears to be a common mechanism by which miRNA efficacy is increased. Such multifaceted targeting was also observed by Uhlmann et al., investigating the EGFR-driven cell cycle pathway in MDA-MB-231 breast cancer cells [35]. Specifically, apart from controlling ErbB3 expression, the negatively acting miRNAs analyzed also targeted Adam17 (Figures 4 and 5D). This metalloproteinase plays an important role in the shedding of ErbB ligands, leading to autocrine and paracrine stimulation of cancer cells, a mechanism often activated in drug resistance [36]. It will be interesting to validate in future studies the suppression of Adam17 at the protein level and investigate its contribution to cell autonomous signaling in the context of the screen hit miRNAs.

We further provide evidence that miR-149, miR-326, miR-148b and miR-520a-3p block HRG-mediated viability of MCF7 cells and thus possess tumor suppressive properties. In support of this, miR-148b was reported to be downregulated in gastric cancers and aggressive breast tumors where it was identified to target multiple genes involved in cell signaling, including PIK3CA [37,38]. The latter regulation, however, appears to depend on the cell type, as miR-148b did not affect PIK3CA expression in MCF7 cells, in accordance with our observations. Similarly, a recent miRNA screen performed in the ErbB2 amplified cell lines KPL-4 and JIMT-1 identified several miRNAs that directly targeted the human ErbB2 UTR [39], none of which were identified by our screen. Considering that the ectopic expression of miR-125a previously reported to target ErbB2 [15], did not affect ErbB2 levels in MCF7 cells suggests that miRNA regulation of this receptor is also highly cell type specific. This may be associated with the fact that the ErbB2 3′UTR does not contain sites that are well conserved amongst mammals [13].

Bioinformatic analysis of the screen data combined with predicted miRNA targets and experimental validation further revealed connections between parallel pathways. The downregulation of Erk1/2 and SOS1 by miR-149, miR-326 and miR-520a-3p plus the predicted targeting of signaling molecules such as FRS2 and Gab1 uncover extensive miRNA-dependent co-regulation of the PI3K and MAPK pathways. Our screen analysis further highlights the interdependence of growth factor and integrin signaling. Rap1a is a small GTPase that is pivotal for integrin activation [40]. Data from our lab shows that miR-149 suppresses Rap1a/b in basal breast cancer cells and impairs cell spreading, migration, and invasion in vitro, and lung metastasis formation in vivo [19]. The coordinate suppression of survival, proliferative and migratory pathways was also seen in the case of miR-148b [37]. Similarly, the miR-520 family was shown to suppress both NFκB and TGFβ signaling in breast cancer cells, providing another example of pathway co-regulation by miRNAs [41].

Of the 24 miRNAs identified to enhance HRG-induced Akt activation, some are likely to possess oncogenic function. The respective miRNA-gene network was less complex and interconnected, reflecting the fact that fewer negative regulators are contained within the ErbB signaling pathway. Thus, apart from the screening data, the reconstructed miRNA-gene networks are influenced by the choice of databases used to link the miRNA targets to signaling pathways. Pathway topology information was retrieved from Reactome, a manually curated and fast growing pathway database, and combined with protein-protein interaction data. For the identification of potential miRNA targets of the screen hits we used three major databases of computationally predicted miRNA targets using different prediction algorithms to ensure finding as many potential targets as possible. The distinct miRNA-gene networks obtained for the positive and negative screen hits confirm the validity of this approach. However, these networks have to be seen within the limitation of the prior knowledge used for their construction. Nevertheless, target validation for the positively acting miRNAs is also of interest as they may regulate the expression of tumor suppressor genes. Intriguingly, PTEN was not identified as a top miRNA-regulated gene by our screen data analysis, although this lipid phosphatase is one of the most important negative regulators of the PI3K pathway. There are reports on PTEN targeting by miRNAs, e.g. miR-21, miR-22, and miR-221/2 [42], but protein loss by mutations, promoter methylation, and LOH may be more common mechanisms of PTEN inactivation. However, because PTEN downregulation increases both basal and HRG-induced Akt activity, miRNAs acting on PTEN may not have been enriched by our analysis filtering those miRNAs that specifically increased Akt activation in response to HRG stimulation. Instead, the phosphatases INPP4A/B and INPP5B involved in phosphoinositide metabolism, and PHLPP2, which directly desphosphorylates Akt, were enriched in our miRNA target gene list, raising the possibility that these phosphatases are potentially more prone to regulation by miRNAs and are particularly involved in determining the amplitude of the HRG response.

In many cases, the miRNAs identified by our screen differentially affected phospho-Akt levels, depending on whether cells were stimulated or not. In our experimental set-up, cells were given three days to adapt to transfection, during which transcriptional changes and feedback mechanisms are likely to impact basal Akt phosphorylation. This differs from the acute stimulation with HRG, for which the expression levels and activity states of the direct upstream regulators of Akt are likely to dominate the response. For example, miR-155 enhanced basal Akt activity, while suppressing HRG-induced Akt activity. miR-155 is an established oncomiR that is upregulated in various cancers including those of the breast. Inhibition of endogenous miR-155 resulted in cell cycle arrest and the induction of apoptosis, whereas ectopic expression promoted survival, proliferation and chemoresistance of breast cancer cells [43]. These results are consistent with a role for miR-155 as a positive regulator of basal PI3K pathway activation. By contrast, miRNAs whose expression reduced basal Akt activity may be important for the control of cell homeostasis and indeed there was a trend toward reduced cell numbers upon transfection of these miRNAs (Additional file 1: Figure S6). However, it should be noted that the alteration of a single pathway is not sufficient to classify a given miRNA as oncogenic or tumor suppressive. For example, in MCF7 cells, members of the miR-520/373 family, which potently suppressed phospho-Akt levels associated with the inhibition of cell proliferation, were reported to promote breast cancer metastasis by targeting CD44 [44]. In ER negative breast cancer cells, however, miR-520c inhibited in vitro cell invasion and in vivo intravasation, and its expression in patient samples was inversely correlated to lymph node metastasis [41]. Together, these observations highlight the fact that miRNAs act in a context-dependent and stage-specific manner.

## Conclusions

Clinical trials have shown the limited efficacy of single-agent Akt pathway inhibitors, presumably due to the activation of compensatory signaling feedbacks [45]. Given their promiscuous target selection, miRNAs may efficiently block such cellular evasion strategies. Furthermore, cancer cells often harbor activating mutations in key pathway molecules, making them resistant to pharmacological inhibition while sensitivity to miRNA regulation should remain. By targeting the Akt pathway at multiple levels, the miRNAs identified in this study may not only be relevant to HRG signaling as Akt is activated downstream of different cancer-promoting RTKs via conserved mechanisms, implying broader clinical applications. Increasing the molecular understanding of miRNA function together with the development of improved delivery systems are prerequisites for the translation of miRNAs to the clinic as anti-cancer therapeutics. The identification and functional characterization of miRNAs targeting the ErbB receptor signaling network and the PI3K pathway in particular are first steps in this direction.

## Methods

### Plasmid constructs

The pGL3-ErbB3 3′UTR firefly luciferase plasmid comprising the first ~700 nucleotides of the ErbB3 3′UTR was kindly provided by Christopher C. Benz [15]. Within the ErbB3 3′UTR, seed regions of miR-149 (527–533) and miR-520a-3p (514–520 and 664–670), respectively, were deleted by site-directed mutagenesis using the pGL3-ErbB3 3′UTR firefly luciferase plasmid as a template and the following forward primers: ErbB3-del-3′UTR-527 forward 5′-CATAATTCAGCACTTAACTATGCATCATACTAAACTTCACC-3′, ErbB3-3′UTR-del-514 forward 5′-GATATTGATTACTATCATAATTCAACTATGAGCCAGGCATC-3′, and ErbB3-3′UTR-del-664 forward 5′-CATGCCTGTAATCTCTGGGAGGCTGAGGC-3′. The ErbB3 mRNA regions 4192–4383 and 5286–5470 (accession no. NM_001982) containing the miR-148b and miR-326 binding sites, respectively, were amplified by PCR using genomic DNA from MCF7 cells as a template and the following primer pairs: ErbB3-4192 forward 5′-TTTTTCTAGACATTATGCCCGCCTAAAAACTCT-3′, ErbB3-4383 reverse 5′- TTTTCTAGAGGGAATAGGGAGAAGACGGTA-3′ and ErbB3-5286 forward 5′- TTTTCTAGATCCTCCCAATTCCTGTGCAT-3′, ErbB3-5470 reverse 5′-TTTTCTAGACCTTCTGACTACCCCCACCA-3′. The PCR products were cloned into the *Xba*I site of the pGL3 vector and, in a second step, the miR-148b (5382–5388) and miR-326 (4292–4298) binding sites were deleted by site-directed mutagenesis using pGL3-ErbB3-4192-4383 and pGL3-ErbB3-5286-5470 as templates and the respective forward primers: ErbB3-del-4292 forward 5′- CCCCAAGGCTAATGAACGTAACTCCTGC-3′, and ErbB3-del-5382 forward 5′-GGCACTGTTTCTTGTTTTAATCAAGTCTAACCCC-3′. Primers were synthesized by MWG, Ebersberg, Germany. Successful cloning and mutagenesis was verified by sequencing (GATC Biotech AG, Konstanz, Germany). The pRL-TK Renilla Luciferase Control Reporter Plasmid was from Promega (Madison, WI, USA).

### Antibodies and reagents

Antibodies used for immunoblotting were: rabbit anti-pErbB2(Y1221/1222) mAb, rabbit anti-pErbB3(Y1289) mAb, rabbit anti-pAkt(T308) mAb, rabbit anti-pAkt(S473) mAb, rabbit anti-pERK1/2(T202/Y204) pAb, mouse anti-ERK1/2 mAb, mouse anti-Akt(pan) mAb, rabbit anti-PI3K p110α mAb (Cell Signaling, Danvers, MA, USA), mouse anti-ErbB2 mAb, mouse anti-ErbB3 mAb (Thermo Scientific, Fremont, MA, USA), and mouse anti-tubulin mAb (Sigma-Aldrich, St Louis, MO, USA). HRP-labeled secondary anti-mouse and anti-rabbit IgG antibodies were from GE Healthcare (Buckinghamshire, UK) and IRDye-680LT anti-rabbit IgG or IRDye-800CW anti-mouse IgG conjugated secondary antibodies were from LI-COR Biosciences (Lincoln, NE, USA). Heregulinβ1 (HRG) was from Peprotech, Rocky Hill, NJ, USA.

### Cell culture and transfection

MCF7 and HEK293T cells were cultured in RPMI 1640 (Life Technologies, Carlsbad, CA, USA), and SKBR3 cells in DMEM (Life Technologies), supplemented with 10% FCS (PAA Laboratories, Cölbe, Germany). Cells were incubated in a humidified atmosphere of 5% CO_2_ at 37°C. Cells were transfected with siRNAs and miRNAs in a final concentration of 10 nM using RNAiMax (Life Technologies) according to manufacturer’s instructions. miRNA-148b, miR-149-5p, miRNA-326, miR-520a-3p, miRNA mimic negative control #1, EGFR-, ErbB3- and ErbB4-SMARTpools were synthesized by Thermo Scientific, Carlsbad, CA, USA. siLacZ (5′-GCGGCUGCCGGAAUUUACC-3’) and siErbB2 (5’-GGACGAAUUCUGCACAAUG-3’) were synthesized by MWG.

### Luciferase assays

4 × 10^4^ HEK293T cells were plated into 96-well plates. The next day, cells were cotransfected with 10 ng wild-type or mutated ErbB3 pGL3 firefly luciferase plasmid, 10 ng pRL Renilla luciferase plasmid, and 50 nM miRNA using Lipofectamine 2000 (Invitrogen). 24 h after transfection, cells were lysed with passive lysis buffer (Promega). The activities of the luciferases were measured by sequential substrate addition as described by Dyer et al. [46] and detection at 562 nm with the multimode reader Infinite® 200 PRO (Tecan). Firefly luciferase activities were normalized to those of Renilla luciferase activities in each sample.

### Quantitative PCR

Total RNA was isolated from 5 × 10^5^-1 × 10^6^ cells using the mirVANA™ miRNA Isolation Kit (Life Technologies) according to the manufacturer’s protocol. Primers for ErbB3, RAP1A and GAPDH were QuantiTect Primer Assays from Qiagen, primers for SOS1 (forward: 5’-CGAGCCCTTTTCACTCAAGC; reverse: 5’-GCCATGGGGCAGAGTAACTT). were designed using PRIMER3 [47,48] and synthesized by biomers.net GmbH, Ulm, Germany. ErbB3, SOS1, RAP1A and GAPDH mRNA levels were quantified by qRT-PCR using the QuantiTect SYBR Green RT-PCR Kit (Qiagen, Foster City, CA, USA) according to manufacturer’s protocol. qRT-PCR was performed with a Cfx96 device (Biorad, Hercules, CA, USA). Changes in the relative expression level were calculated using the 2^-ΔΔCt^ method (Biorad CFX manager software 2.1.). GAPDH was used as the endogenous control gene.

### Cell lysis, SDS-PAGE and Western Blotting

Cells were lysed in RIPA buffer [50 mM Tris (pH 7.5), 150 mM NaCl, 1% Triton-X-100, 0.5% sodium deoxycholate, 0.1% SDS, 1 mM sodium orthovanadate, 10 mM sodium fluoride, and 20 mM β-glycerophosphate plus Complete protease inhibitors (Roche, Basel, Switzerland)] and lysates were clarified by centrifugation at 16,000 × g for 10 min. Proteins where separated by SDS-Page and transferred to a Nitrocellulose membrane using the iBlot Gel Transfer Device (Life Technologies). The membrane was blocked with 0.5% blocking reagent (Roche) in PBS containing 0.1% Tween-20 and then incubated with primary antibodies, followed by HRP- or IRDye-conjugated secondary antibodies. Visualization was carried out using the ECL detection system (Thermo Fischer) or the Odyssey device (LI-COR Bioscience).

### miRNA library screening

7,5 × 10^4^ cells/well were seeded into 96-well plates and transfected with the miRNA library (Thermo Fischer Scientific, see Additional file 2: Table S1) comprising 879 miRNA mimics. The RNA was transfected in a final concentration of 10 nM using Lipofectamine RNAiMax according to manufacturer’s instructions (Life Technologies) in two biological replicates. 72 h after transfection the cells were either stimulated with 10 ng/ml HRG for 1 h or left untreated, followed by In-Cell Western analysis. The miRNA library was segmented into 11 × 96-well plates, each plate containing 80 miRNA mimics, 2 negative controls (siLacZ and miR-con) and 3 positive controls (siErbB2, siErbB3 and miR-149-5p).

### In Cell Western analysis

Cells were fixed with 4% PFA for 20 min, permeabilized with PBS containing 0.1% Triton X-100 for 5 min and washed twice with PBS containing 0,1% Tween 20. Unspecific binding was blocked with 0.5% blocking reagent (Roche) in PBS containing 0.1% Tween-20. Cells were then incubated with primary antibodies, followed by IRDye-conjugated secondary antibodies. Visualization was carried out with the Odyssey device and images were processed with the Odyssey software version 2.1. Finally, plates were stained with crystal violet to monitor cell density. Crystal violet was dissolved in methanol and measured at 550 nm with the multimode reader Infinite® 200 PRO (Tecan).

### Screen data analysis

All statistical and bioinformatic analyses were performed using R statistical computing environment (http://www.R-project.org) [49]. First, raw data of each 96-well plate was corrected for the signal decay of pAkt caused by the successive PFA fixation of the individual columns. This was done by comparing the medians of the first and the last column per plate harboring the same set of controls, which theoretically should have identical median values. Assuming a linear signal decay, a correction value for each column was calculated and added to the corresponding column. For the corrected values, the ratio of pAkt/Akt was calculated resulting in the value for Akt activity. Next, the ratios were log2-transformed. HRG-induced Akt activation was calculated by subtracting the pAkt/Akt ratio for basal, unstimulated cells from that of HRG-stimulated cells, yielding the increase in Akt activation upon HRG stimulation (ΔpAkt). Two-sided *t*-test was applied to compare ΔpAkt of each miRNA in the screen against negative controls and resulting p-values were adjusted for multiple comparisons using Benjamini & Hochberg correction [50]. miRNAs with adjusted p-value < 0.05 were considered as significant.

### Viability assays

One day after transfection, 2.5 × 10^3^ cells were plated into 96-well plates. The next day, medium was replaced by medium containing 0.5% FCS ± 5 ng/ml HRG. Five days later, cells were fixed with paraformaldehyde and stained with crystal violet. Crystal violet was dissolved in methanol and measured at 550 nm with the multimode reader Infinite® 200 PRO (Tecan).

### Migration assays

Three days after transfection, SKBR3 cells (1 × 10^5^) were seeded in medium containing 0.5% FCS into Transwells (8 μm pore size; Sigma Aldrich, St. Louis, MI, USA) coated with 10 μg/ml collagen (Serva, Germany) on the underside and allowed to migrate overnight. The bottom chamber contained medium with 0.5% FCS supplemented with 25 ng/ml HRG. Cells on the underside of the membranes were fixed, stained with crystal violet, and counted in five independent microscopic fields. Experiments were performed with duplicate filters.

### Availability of supporting data

The data sets supporting the results of this article are included within the article and its additional files.

## Additional files

Additional file 1: Figure S1. In-Cell-Western establishment. MCF-7 cells were stimulated with different HRG concentrations for different times and analyzed by In-Cell-Western using antibodies specific for (A) Akt(pT308) and Akt, and (B) pErk1/2 and total Erk1/2. Signals were background subtracted and normalized to the unstimulated control. **Figure S2.** Distribution of ΔpAkt values for all positive and negative controls. ΔAkt values <1 indicate a reduction, values >2 an increase in Akt activation upon HRG stimulation. Data analysis: one way Anova followed by Bonferroni’s multiple comparison test (****p* < 0.001, ***p* < 0.01). **Figure S3.** miRNAs and their targets (see Figure 4). The most frequently targeted genes for miRNAs that significantly reduced Akt activity are shown. **Figure S4.** Regulatory miRNA/target network for negatively acting miRNAs. miRNAs (blue); genes (grey circles); miRNA-target interactions (black lines); positive regulatory effects (green lines). The network includes 19 miRNAs and 14 genes targeted by at least 9 miRNAs. Akt1 and Akt2 were included manually. **Figure S5.** Regulatory miRNA/target network for positively acting miRNAs. miRNAs (blue); genes (grey circles); miRNA-target interactions (black lines); positive regulatory effects (green lines). The network includes 21 miRNAs and 17 genes targeted by at least 6 miRNAs. Akt1 and Akt2 were included manually. **Figure S6.** Correlation of miRNA expression with cell density. pAkt/Akt ratios and crystal violet values were correlated for the unstimulated (basal) condition. **Figure S7.** Predicted binding sites of miR-148b, miR-326 and miR-520a in the ErbB3 mRNA. **Figure S8.** miRNA inhibition of HRG signaling in SKBR3 cells. Cells were transfected with the indicated miRNAs. (A) Lysates from untreated (0 min) and HRG-stimulated cells were analyzed by immunoblotting with the indicated antibodies Cropped panels are from the same blot. (B) Transwell assays with HRG as a chemotactic stimulus. The mean ± SEM of two (miR-149) or three (miR-520a-3p) independent experiments is shown. Additional file 2: Table S1. Sequences contained in the human mimic library. **Table S2.** Raw data for basal and HRG-stimulated cells. **Table S3.** Lists of computationally predicted miRNA targets.
