# Supplementary material for: A global microRNA screen identifies regulators of the ErbB receptor signaling network
Source: Cell Commun Signal. 2015 Jan 29;13:5. doi: 10.1186/s12964-015-0084-z (PMC4314810; doi:10.1186/s12964-015-0084-z)
Supplement: Additional file 1: Figure S1. — In-Cell-Western establishment. MCF-7 cells were stimulated with different HRG concentrations for different times and analyzed by In-Cell-Western using antibodies specific for (A) Akt(pT308) and Akt, and (B) pErk1/2 and total Erk1/2. Signals were background subtracted and normalized to the unstimulated control. Figure S2. Distribution of ΔpAkt values for all positive and negative controls. ΔAkt values <1 indicate a reduction, values >2 an increase in Akt activation upon HRG stimulation. Data analysis: one way Anova followed by Bonferroni’s multiple comparison test (***p < 0.001, **p < 0.01). Figure S3. miRNAs and their targets (see Figure 4). The most frequently targeted genes for miRNAs that significantly reduced Akt activity are shown. Figure S4. Regulatory miRNA/target network for negatively acting miRNAs. miRNAs (blue); genes (grey circles); miRNA-target interactions (black lines); positive regulatory effects (green lines). The network includes 19 miRNAs and 14 genes targeted by at least 9 miRNAs. Akt1 and Akt2 were included manually. Figure S5. Regulatory miRNA/target network for positively acting miRNAs. miRNAs (blue); genes (grey circles); miRNA-target interactions (black lines); positive regulatory effects (green lines). The network includes 21 miRNAs and 17 genes targeted by at least 6 miRNAs. Akt1 and Akt2 were included manually. Figure S6. Correlation of miRNA expression with cell density. pAkt/Akt ratios and crystal violet values were correlated for the unstimulated (basal) condition. Figure S7. Predicted binding sites of miR-148b, miR-326 and miR-520a in the ErbB3 mRNA. Figure S8. miRNA inhibition of HRG signaling in SKBR3 cells. Cells were transfected with the indicated miRNAs. (A) Lysates from untreated (0 min) and HRG-stimulated cells were analyzed by immunoblotting with the indicated antibodies Cropped panels are from the same blot. (B) Transwell assays with HRG as a chemotactic stimulus. The mean ± SEM of two (miR-149) or three (miR-520a-3p) indepen [file 12964_2015_84_MOESM1_ESM.pdf]

Figure S1

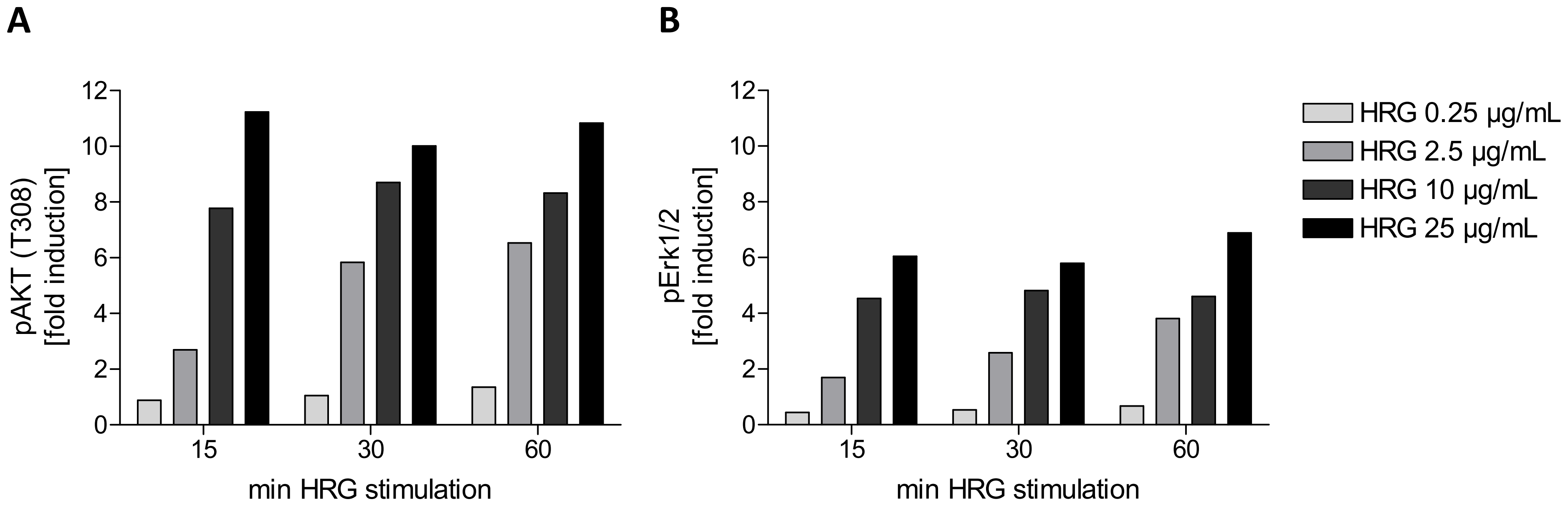
A
B
12
10
8
6
4
2
0
pAKT (T308)
[fold induction]
15
30
60
min HRG stimulation
pErk1/2
[fold induction]
HRG 0.25 µg/mL
HRG 2.5 µg/mL
HRG 10 µg/mL
HRG 25 µg/mL

Figure S2

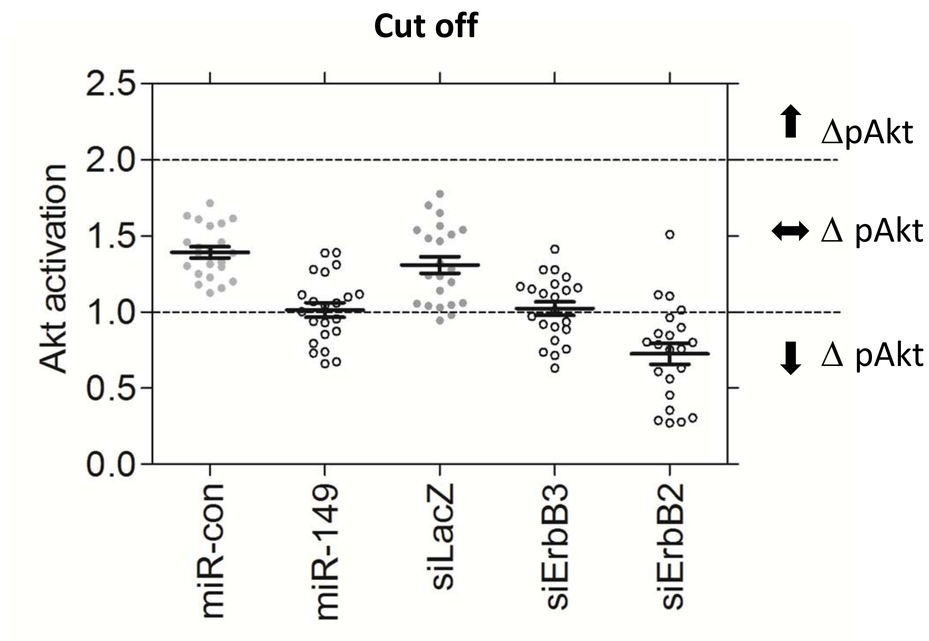

Bonferroni's Multiple Comparison Test	
miR-con vs miR-149	***
miR-con vs siLacZ	ns
miR-con vs siErbB3	***
miR-con vs siErbB2	***
miR-149 vs siLacZ	***
miR-149 vs siErbB3	ns
miR-149 vs siErbB2	**
siLacZ vs siErbB3	**
siLacZ vs siErbB2	***
siErbB3 vs siErbB2	***

Figure S3

	miR-19a	miR-148b	miR-149	miR-155	miR-204	miR-302c	miR-326	miR-520a-3p	miR-520d-3p
SOS1	X	X			X	X	X	X	X
RPS6KA5	X	X			X	X		X	X
PDE4D	X	X	X	X	X	X		X	X
RAP1A	X	X	X	X		X		X	X
mTORC2	X	X		X	X		X		
EGFR		X	X	X	X	X		X	X
APBB1IP				X	X	X	X	X	X
ADAM17		X	X		X	X	X	X	X
CPA3	X	X		X		X		X	X
FRS2	X	X		X	X	X		X	X
CDKN1B		X	X	X		X		X	X
CALD1	X	X	X		X	X		X	X
ERBB3	X	X	X	X	X	X	X	X	X
PIK3	X	X	X	X	X	X		X	X

Figure S4

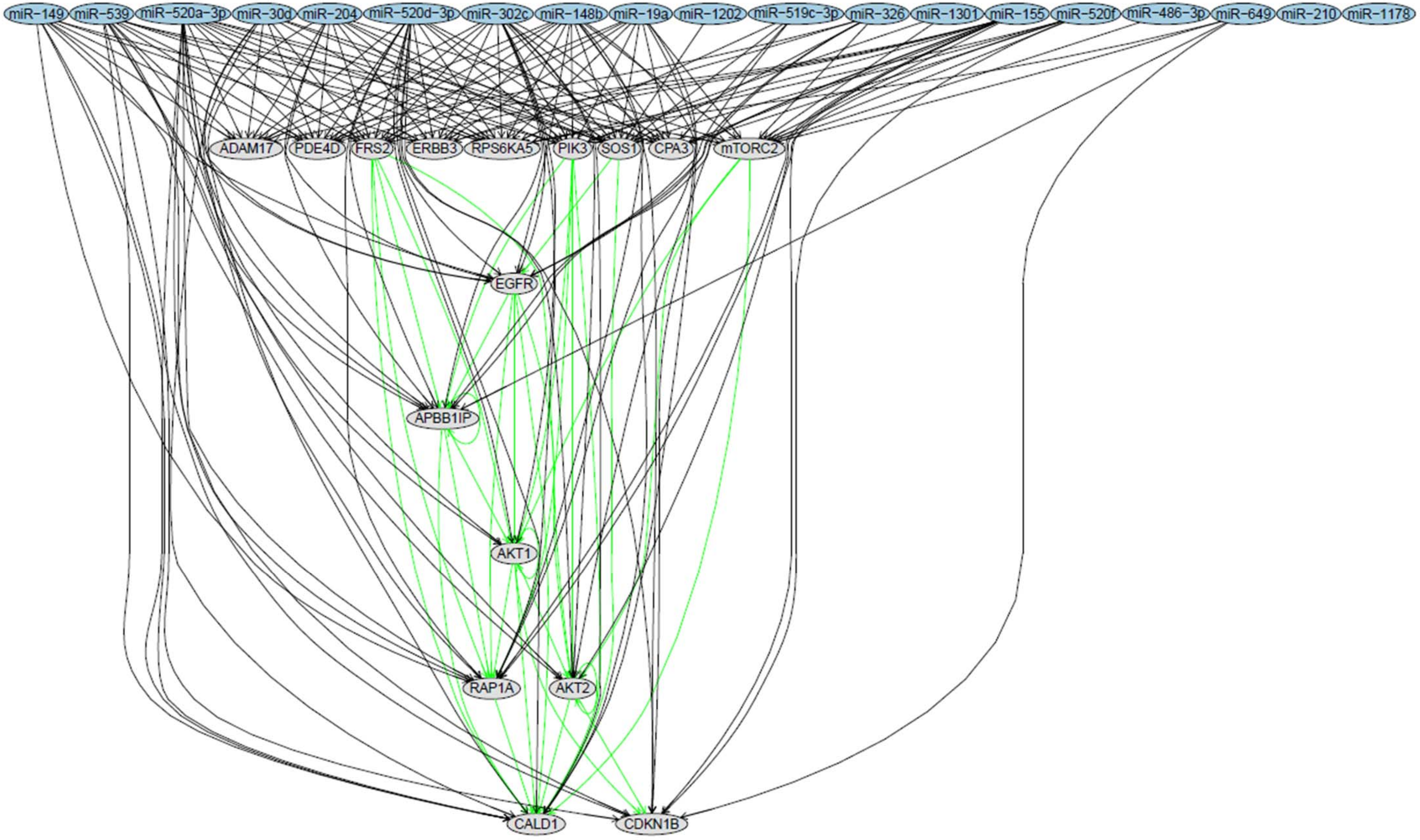

miR-149
miR-539
miR-520a-3p
miR-30d
miR-204
miR-520d-3p
miR-302c
miR-148b
miR-19a
miR-1202
miR-519c-3p
miR-326
miR-1301
miR-155
miR-520f
miR-486-3p
miR-649
miR-210
miR-1178
ADAM17
PDE4D
FRS2
ERBB3
RPS6KA5
PIK3
SOS1
CPA3
mTORC2
EGFR
APBB1IP
AKT1
RAP1A
AKT2
CALD1
CDKN1B

Figure S5

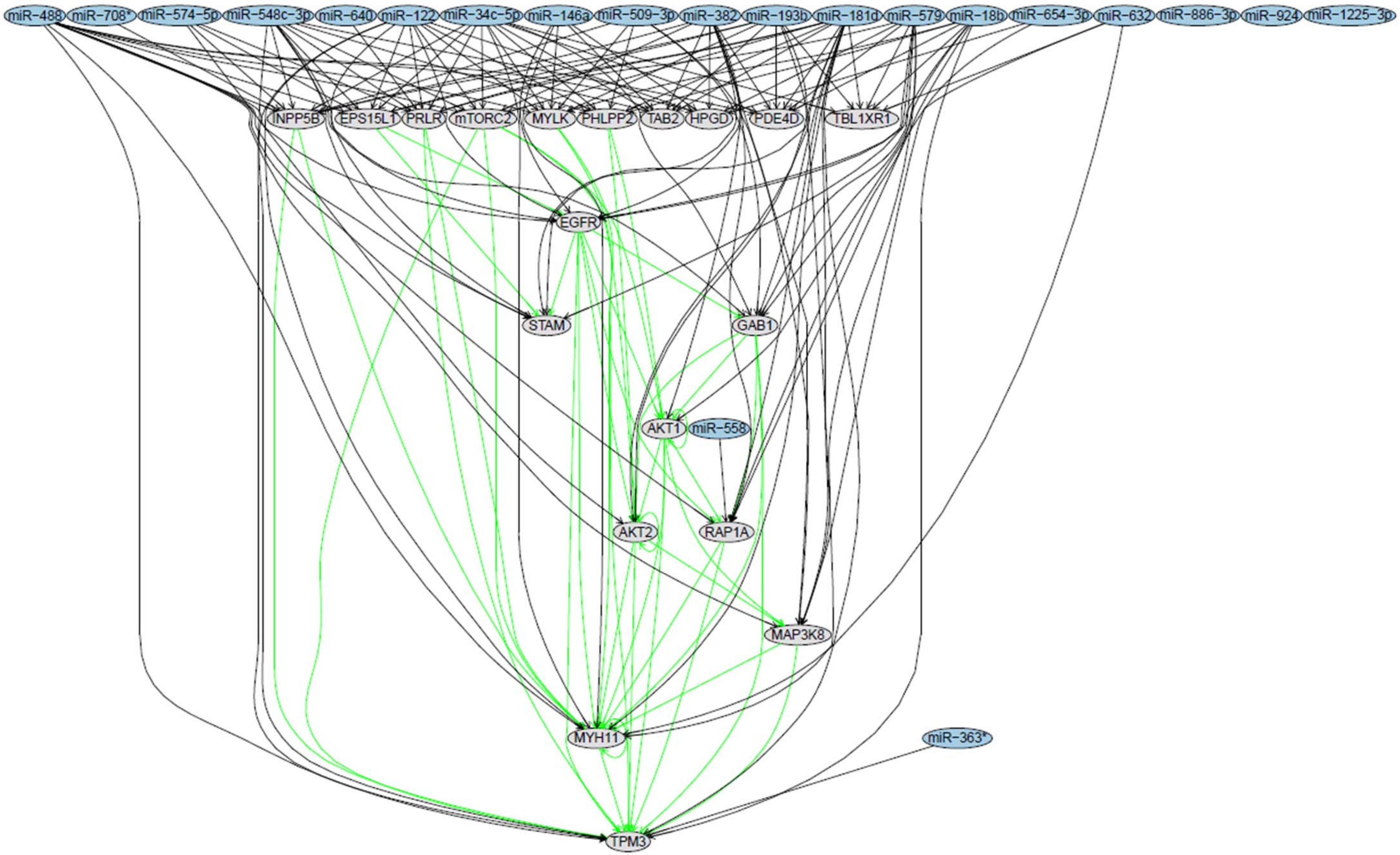

miR-488
miR-708*
miR-574-5p
miR-548c-3p
miR-640
miR-122
miR-34c-5p
miR-146a
miR-509-3p
miR-382
miR-193b
miR-181d
miR-579
miR-18b
miR-654-3p
miR-632
miR-886-3p
miR-924
miR-1225-3p
INPP5B
EPS15L1
PRLR
mTORC2
MYLK
PHLPP2
TAB2
HPGD
PDE4D
TBL1XR1
EGFR
STAM
GAB1
AKT1
miR-558
AKT2
RAP1A
MAP3K8
MYH11
miR-363*
TPM3

Figure S6

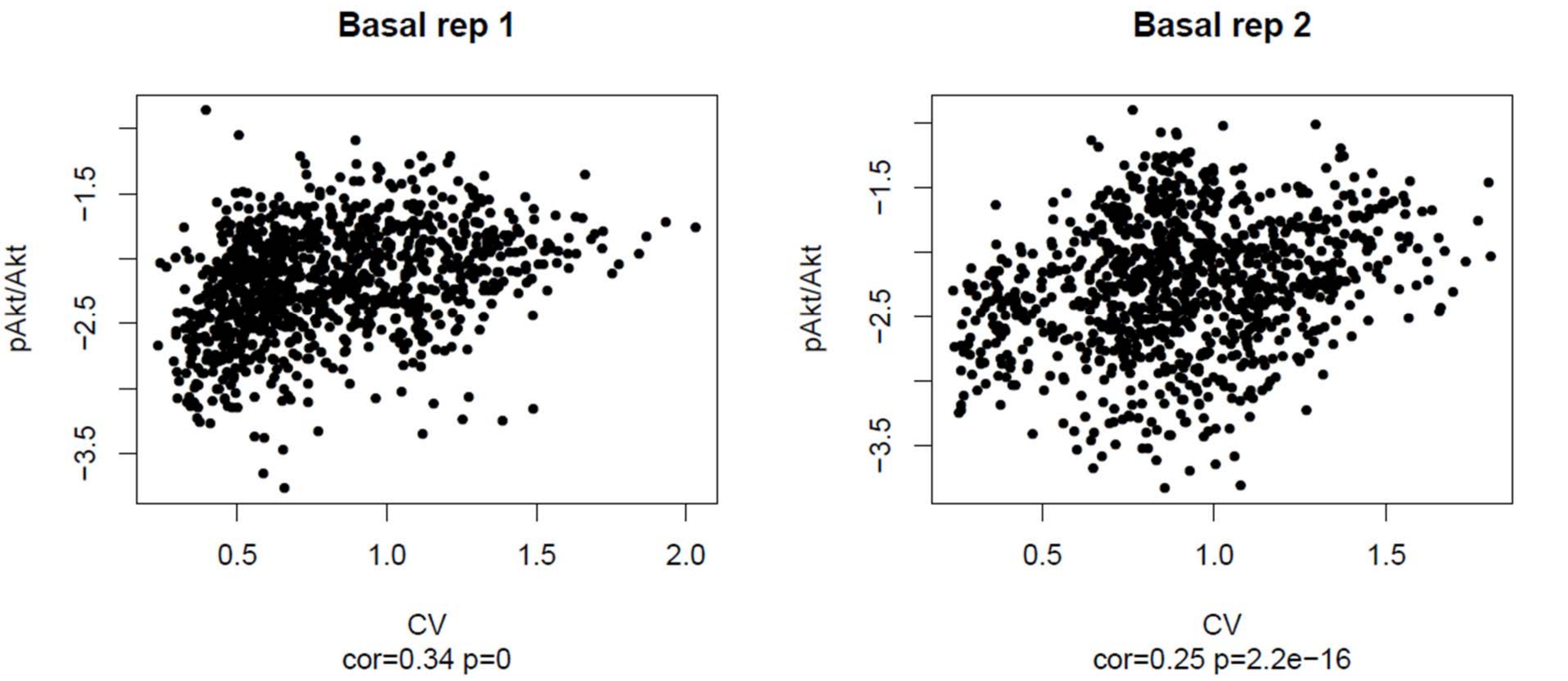
Basal rep 1
pAkt/Akt
−1.5
−2.5
−3.5
0.5
1.0
1.5
2.0
CV
cor=0.34 p=0
Basal rep 2
pAkt/Akt
−1.5
−2.5
−3.5
0.5
1.0
1.5
CV
cor=0.25 p=2.2e−16

Figure S7

pGL3-ErbB3 3’UTR (Scott et al., 2007)

hsa-miR-520a-3p / ERBB3 alignment

```
     3‘ ugucaGGUUUCCCUUCGUGAAa 5‘ hsa-miR-520a-3p
             ¦|| |    |||||||
500: 5‘ uacuaUCAUAAUUCAGCACUUa 3‘ ERBB3

     3‘ ugucagguuucccuUCGUGAAa 5‘ hsa-miR-520a-3p
                      |||||||
650: 5‘ augccuguaaucucAGCACUUu 3‘ ERBB3
```

ErbB3 mRNA (NM_001982)

hsa-miR-148b / ERBB3 alignment

```
     3‘ uguuucAAG-AC-ACUACGUGACu 5‘ hsa-miR-148b
              ||| || |  |||||||
5366:5‘ cacuguUUCUUGUUUUUGCACUGa 3‘ ERBB3
```

hsa-miR-326 / ERBB3 alignment

```
     3‘ gaccUCC-UUCCCGGGUCUCc 5‘ hsa-miR-326
            |||  |  ||||||||
4279:5‘ cccaAGGCUAAUGCCCAGAGa 3‘ ERBB3
```

Figure S8

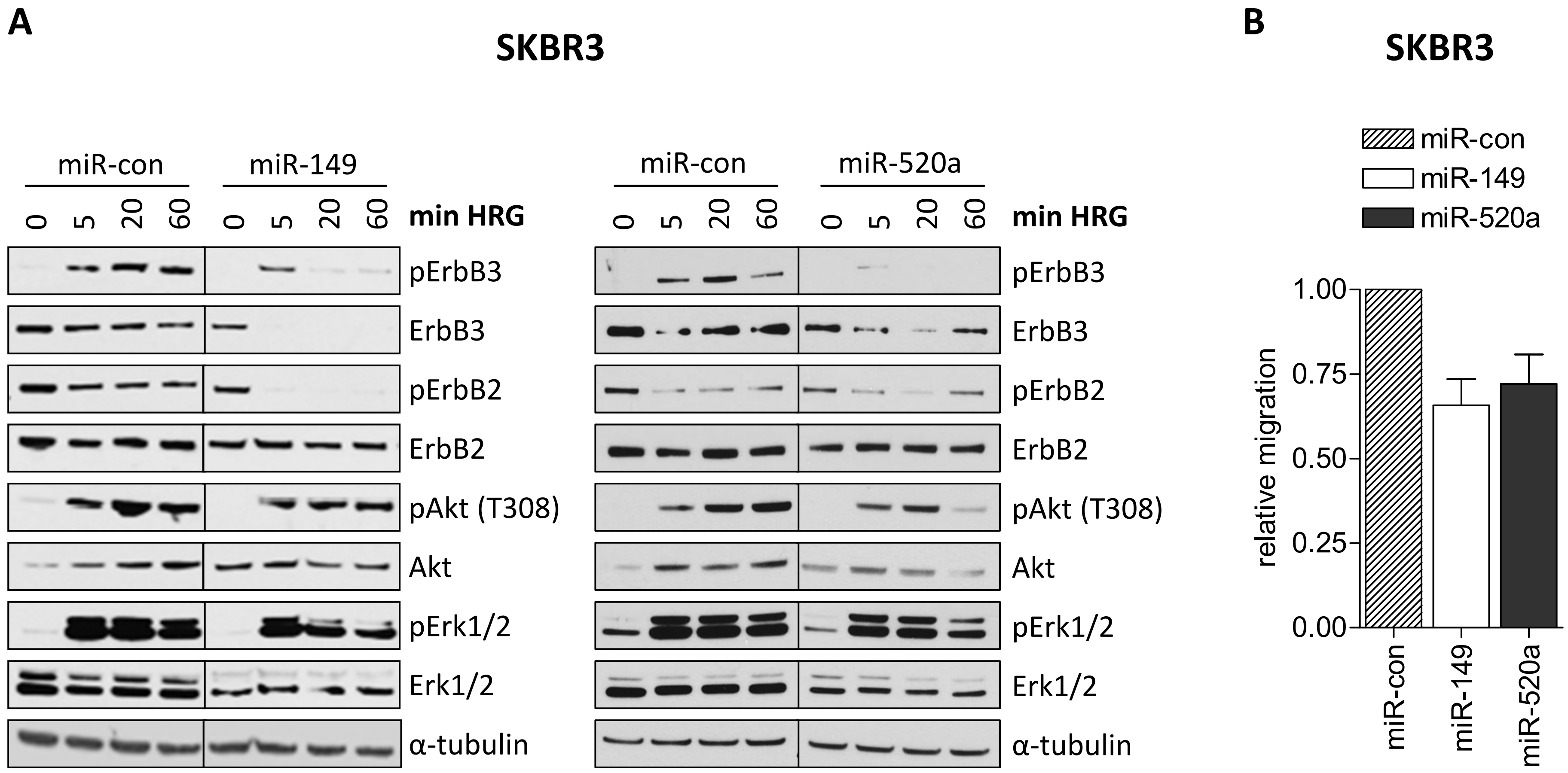

A
SKBR3
miR-con
miR-149
miR-con
miR-520a
0 5 20 60 0 5 20 60 min HRG
pErbB3
ErbB3
pErbB2
ErbB2
pAkt (T308)
Akt
pErk1/2
Erk1/2
α-tubulin
B
SKBR3
miR-con
miR-149
miR-520a
relative migration
1.00
0.75
0.50
0.25
0.00
